# THE ASSOCIATION OF EARLY LIFE ADVERSITY AND LIFE COURSE RELATIONSHIP QUALITY WITH LATE-LIFE COGNITIVE HEALTH

**DOI:** 10.1093/geroni/igae098.4353

**Published:** 2024-12-31

**Authors:** Monica Walters, Roger Wong, Kiana Scambray, Toni Antonucci, Wassim Tarraf

**Affiliations:** University of Michigan, Ann Arbor, Michigan, United States; State University of New York Upstate Medical University, Syracuse, New York, United States; University of Michigan, Ann Arbor, Michigan, United States; University of Michigan, Ann Arbor, Michigan, United States; Wayne State University, Detroit, Michigan, United States

## Abstract

Early life adversity (ELA) relates negatively to cognition, yet how particular ELA experiences may differentially relate to cognition and whether associations may be attenuated by life course relationship quality (LCRQ) is unclear. We aimed to assess how ELA subgroups related to cognition and whether associations may be buffered by LCRQ. Adults (ages 50-64 at baseline) from the Health and Retirement Study participated (n=4,193; 2006/2008=baseline; 2018/2020=follow-up). Latent class and profile analyses identified ELA and LCRQ subgroups, respectively. We related ELA and LCRQ subgroups to global cognition (linear regression) as well as risk of cognitive impairment, not dementia (CIND) and dementia (multinomial logistic regression) at follow-up. We included sociodemographic and health-related covariates. We identified 4 ELA (High Adversity, Family Disruptions, Elevated Household Trauma, Low Adversity) and 3 LCRQ (Strong Ties, Mixed Ties, Weak Ties) classes. Of note, racially/ethnically minoritized adults were more likely to belong to Family Disruptions and Weak Ties classes than White adults. Participants with Family Disruptions (vs. Low Adversity) had worse cognition (global: b=-0.84, 95% CI[-1.25;-0.43]; CIND: RRR=1.54, 95% CI[1.16;2.03]; dementia: RRR=1.64, 95% CI[1.02;2.64]); controlling for LCRQ and sociodemographics attenuated associations. Participants with Weak Ties (vs. Strong) had worse cognition (b=-2.70, 95% CI[-3.40;-2.01]) and higher risk of CIND (RRR=3.22, 95% CI[2.11;4.89]) and dementia (RRR=4.10, 95% CI[2.16;7.81]); associations were not explained by covariates. Family Disruptions negatively impacted cognition, but associations were explained by sociodemographics, indicating potential resilience from ELA. However, negative LCRQ, particularly Weak Ties, was consistently harmful to cognition. Therefore, targeting midlife and late-life social relationships may benefit cognition.

